# Case Report: Lumbar spondylolisthesis with unilateral pedicle cleft and contralateral spondylolysis: a report of two cases and literature review

**DOI:** 10.3389/fsurg.2024.1357282

**Published:** 2024-06-28

**Authors:** Sheng Chang, Yu Wang, Yong Liu, Chao Wang

**Affiliations:** ^1^ Department of Spine Surgery, The Affiliated Hospital of Qingdao University, Qingdao, China; ^2^ Department of Surgery, Kashgar Hospital of Chinese Medicine, Kashgar, the Xinjiang Uygur Autonomous Region, China

**Keywords:** pedicle cleft, lumbar spondylolisthesis, spondylolysis, stress fracture, dysplasia

## Abstract

**Background:**

The causes of pedicle cleft include congenital dysplasia and stress fractures, both of which are rare conditions. Secondary lumbar spondylolisthesis with combined unilateral pedicle cleft and contralateral spondylolysis is extremely rare and can be easily misdiagnosed. We report two cases with these conditions from different causes and discuss the diagnostic and therapeutic features in the context of the literature review.

**Case description:**

Case 1 was a 58-year-old female with a stress fracture change at the left L5 pedicle. Case 2 was a 47-year-old female with a pedicle cleft due to hypoplasia of the left L5 pedicle. Both patients had a combined contralateral spondylolysis and Meyerding grade one lumbar spondylolisthesis, while neither had a clear history of lumbar trauma. After initial conservative treatments failed, both patients underwent a single-segment posterior lumbar interbody fusion with bilateral pedicle screw fixation. Both patients were followed up for more than 1 year postoperatively with clinical symptom relief and bony fusion at the pedicle cleft suggested by a CT scan.

**Conclusion:**

Lumbar spondylolisthesis with unilateral pedicle cleft and contralateral spondylolysis is rarely reported and can be clinically misdiagnosed as simple spondylolisthesis with bilateral spondylolysis. There is no widely accepted surgical option for patients for whom conservative treatment has failed. Our experience suggests that good clinical results may be achieved by single-segment posterior interbody fusion and bilateral pedicle screw fixation. Precise screw placement into the deficient pedicle and sufficient exiting nerve decompression are prerequisites for the success of this surgical option.

## Introduction

The causes of pedicle cleft include congenital pedicle dysplasia and cleft caused by continuous stress fracture pediculolysis (1–13). Pedicle dysplasia is a rare developmental deformity, while the involvement of the lumbar or sacral vertebrae is rare (3, 7, 12). Stress fracture of the pedicle was named by Gunzburg and Fraser as “pediculolysis,” in which pedicle hypertrophy, sclerosis, pseudoarthrosis, and bone nonunion are indicated in a stress-persisting microenvironment (1, 2, 4, 8–11, 13). Unilateral pedicle cleft combined with contralateral spondylolysis is a rare cause of lumbar spondylolisthesis and is likely misdiagnosed as more common bilateral spondylolysis. Herein, we report two cases of pedicle cleft (one caused by congenital dysplasia and the other by pediculolysis) with a combined contralateral spondylolysis and spondylolisthesis. We further review and summarize the literature for a better understanding of the etiology, anatomical features, diagnosis, and surgical options, taking together to provide useful references for surgeons encountering similar rare cases.

## Case description

### Case 1

**Figure 1:**
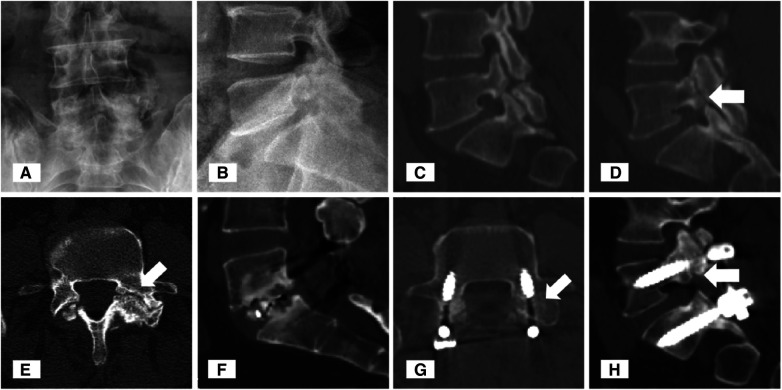
Typical images of Case 1. An anteroposterior plain radiograph revealed the obscure boundary of the left L5 pedicle (**A**). Lateral plain radiograph revealed Meyerding grade 1 spondylolisthesis (**B**). Sagittal and axial computed tomography (CT) images of the lumbar spine showed right spondylolysis (**C**) and right pediculolysis [(**D**,**E**), white arrowheads] of the L5 vertebra. Postoperative CT demonstrated that bony fusion occurred at the L5-S1 interbody space (**F**) and at the preoperative site of pedicle cleft (**G**,**H**).

**Figure 2:**
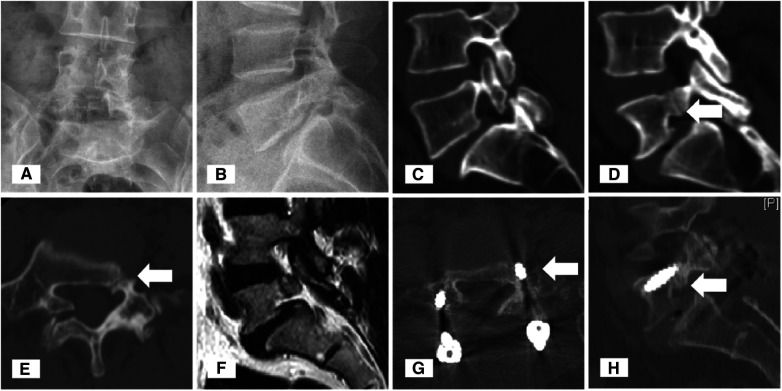
Typical images of Case 2. An anteroposterior plain radiograph revealed the obscure boundary of the L5 left pedicle (**A**). Lateral plain radiograph revealed Meyerding grade 1 spondylolisthesis (**B**). Sagittal and axial computed tomography (CT) images of the lumbar spine showed right spondylolysis (**C**) and right dysplasia (**D**,**E**) and the pedicle cleft (white arrowheads) of the L5 vertebra. T2-weighted sagittal magnetic resonance imaging of the left pedicle showed a hypointense signal of the cleft (**F**). Postoperative CT demonstrated that bony fusion occurred at the preoperative pedicle cleft (**G**,**H**) site.

A 58-year-old female who was a farmer complained of low back pain for six months, and it worsened progressively one month ago. The patient received medication and rest for 3 months, but the effect was unsatisfactory. On examination, the range of motion (ROM) of the lumbar spine was decreased and interspinous tenderness was positive at the L5-S1 level. Both sensation and muscle strength of the lower limbs are normal. The visual analog scale (VAS) score was eight points for low back pain. Anteroposterior and lateral x-rays showed poor visualization of the left L5 pedicle and a Meyerding grade 1 spondylolisthesis at the L5, respectively (Figures 1A,B). CT showed a right spondylolysis of L5 and a left pediculolysis at the same vertebra with surrounding hyperostosis and sclerosis, especially at the edges of the pedicle defect area (Figures 1C–E). Magnetic resonance imaging (MRI) showed hypointense on both T1- and T2-weighted images at the cleft area. She underwent intervertebral fusion and concurrent bilateral pedicle screw fixation at the L5-S1 level. Postoperatively, her back pain resolved and the VAS score was two points at her discharge. At the 2-year follow-up, sagittal and axial views on the CT scan showed signs of intervertebral fusion and fusion of the pedicle cleft and the fine screw position (Figures 2F–H).

### Case 2

A 47-year-old female patient who was also a farmer suffered from lower back pain and radiating pain in the lower limbs for 1 year. She also depicted typical neurogenic claudication which could be relieved by immediate rest. Previously, she underwent physiotherapy for 6 months, but the pain became intractable recently. On physical examination, interspinous and paravertebral tenderness was positive at the L5-S1 level, while normal sensation and muscle strength were recorded at the lower limbs. The VAS score was seven points for the back pain and six points for the leg. X-rays showed Meyerding grade 1 spondylolisthesis of the L5, poor visualization of the left L5 pedicle, and spondylolysis on the right side (Figures 2A,B). CT showed a hypoplastic change of the left L5pedicle, a defect at the junction between the pedicle and vertebral body, and a minor hyperplastic change on the margin (Figures 2C–E). MRI showed hypointense of the pedicle cleft on both T1- and T2-weighted images without surrounding inflammatory changes (Figure 2F). She underwent a posterior approach L5-S1 intervertebral fusion with pedicle screw fixation. Postoperative follow-up showed satisfactory relief of low back and leg pain. At 1 year follow-up, CT showed a well-positioned internal fixation and a trend toward fusion at the pedicle cleft (Figures 2G,H).

## Discussion

Lumbar spondylolysis is well documented, and isthmic spondylolisthesis is one of the major types of lumbar spondylolisthesis. However, the etiology is of debate (1, 4). It is postulated that genetic and developmental factors play important roles. The fusion process of two independent ossification centers is blocked during development, which further leads to spondylolysis (14). On the other hand, it has been proved that the incidence of spondylolysis increases with age and is more likely to occur in young athletes who are subjected to repeated lumbar hyperextension, thus sustained and repeated stress fracture is important for the formation of isthmic defect (1, 4, 15, 16). Stress fracture-induced spondylolysis has certain anatomical and biomechanical evidence. The facet joint orientation of the lower lumbar spine tends to be coronal, and the isthmus is anatomically the weakest part of the vertebral arch, which is subject to the most significant shear and torsional forces (13, 17). Unilateral spondylolysis is less common. According to the literature, the incidence of unilateral lumbar spondylolysis is about one-fifth compared with that of bilateral spondylolysis. The chance of developing spondylolisthesis is even lower, accounting for about one-tenth of the patients with bilateral spondylolysis (18).

Lumbar pedicle stress fracture rarely occurs in clinics because of the short force arm, thick diameter, and great intrinsic strength of the lumbar pedicle (8, 9, 11). Pedicle stress fracture occurs mainly in manual workers and athletes who bear repetitive flexion, extension, or rotation of the lumbar spine. The majority of reported unilateral stress fractures of the lumbar pedicle are on the right side, which suggests the association with habitual right-sided force during throwing or trunk rotational activities (10). It is revealed that in patients with unilateral spondylolysis, the contralateral pedicle often presents with sclerosis or even fracture, a compensatory reaction to the focal overload (2, 5, 6). After studying the CT manifestations of 13 athletes with unilateral spondylolysis, Sakai et al. (17) found that two of them had contralateral pedicle fractures and four had significant contralateral pedicle sclerosis. A further finite element analysis showed that in the presence of unilateral spondylolysis, the stress on the contralateral pedicle and isthmus by axial rotation increased as heavily as 6.8 times on average compared to the situation without unilateral spondylolysis. Thus, although unilateral lumbar spondylolysis usually shows a benign prognosis, it may be a precursor to a contralateral pedicle fracture (10). In 1991, Gunzburg et al. (1) referred to the stress pedicle fracture as “pediculolysis,” the important pathological and imaging features of which are minor, yet persistent trauma resulting in pedicle hypertrophy, sclerosis, and pseudarthrosis, as shown in our Case 1. The other situation in which the pedicle cleft occurs is based on pedicle dysplasia. Pedicle is one of the predilection sites of retrosomatic clefts during embryonic development, with reported segments ranging from T12 to S1 (3, 5, 12, 19). Congenital hypoplasia of the lumbosacral junction can lead to facet joint dysplasia, which has an important impact on local biomechanical stability (3). In addition, it is not uncommon in older people to have hypoplasia-associated pedicle clefts, most of whom have risk factors such as osteoporosis, compression fractures, laminectomy, fusion of adjacent segments, and long-term steroid use (2). It is important to emphasize that in the case of contralateral spondylolysis, there is also localized stress concentrated on the hypoplastic pedicle, so limited hyperplasia and sclerosis may also be presented around the cleft (Case 2), but they are generally not as pronounced as in pediculolysis.

Patients with unilateral spondylolysis may experience spinous tilt, suggesting segmental rotation instability and disc degeneration (1, 20). Segmental instability is more conspicuous when the unilateral spondylolysis is combined with the contralateral pedicle cleft, and the patient may have significant low back pain and limitation of lumbar ROM. The persistence of segmental instability and axial shearing load can progressively lead to the development of spondylolisthesis. Most reported cases with unilateral spondylolysis combined with contralateral pedicle cleft but without spondylolisthesis are adolescents and athletes under 30 years old. To our best knowledge, so far in the literature, there are only six cases (Table 1) who can be diagnosed with spondylolisthesis with concurrent unilateral pedicle cleft and contralateral spondylolysis, including the two patients we presented here (two cases with dysplasia and four cases with pediculolysis). The mean age of the patients was 49.5 years, suggesting that the progression of spondylolisthesis to a significant and symptomatic grade requires considerable load and prolonged time. It is important to note that although this rare cause of spondylolisthesis shares similar biomechanical and pathophysiological mechanisms to the more common spondylolisthesis with bilateral spondylolysis, the two entities should be carefully differentiated, especially in cases where surgical treatment is indicated. In contrast to weight-bearing anteroposterior and lateral x-rays, CT (especially 3D CT) can provide much more detailed information such as the morphology of pedicle cleft, coexisting hyperplasia/hypoplasia, sclerosis, and abnormalities of the pedicle, which is essential for the design of the surgical plan. MRI is valuable in differentiating other conditions as it clearly shows the state of disc degeneration, nerve compression, and signals of soft tissue and bone surrounding the cleft. Compared to CT scans, MRI takes advantage of the detection of isthmus and pedicle osseous edema, which is a sign earlier than the incomplete cortex stress fracture (6).

**Table 1:** Literature summary of spondylolisthesis with unilateral pedicle cleft and contralateral spondylolysis.

Authors, year	Age (years), sex	Level	Meyerding grade	Type of pedicle cleft	Clinical presentation	Image findings	Treatment	Follow-up
Hsieh et al. 2017 (3)	63, male	L4	1	Hypoplasia	LBP and radiculopathy	Right pedicle defect and left spondylolysis	ALIF and L4–5 left percutaneous screw fixation	2 years
Viswanathan et al. 2020 (2)	41, female	L5	1	Pediculolysis	LBP and radiculopathy	Left pedicle defect and right spondylolysis	TLIF with bilateral pedicle screw fixation at L5-S1; revision surgery extended to L4 with left L5 medial pediculectomy and screw removed	2 years
Akhaddar, 2020 (5)	54, male	L5	1	Pediculolysis	Radiculopathy	Right pedicle defect and bilateral spondylolysis	Spinal immobilization, weight loss, and physical therapy	4 years
Kim et al. 2006 (4)	34, male	L5	1	Pediculolysis	LBP and radiculopathy	Right pedicle defect and left spondylolysis	TLIF with bilateral pedicle screw fixation	NM
Current report	58, female	L5	1	Pediculolysis	LBP	Left pedicle defect and right spondylolysis	TLIF with bilateral pedicle screw fixation	2 years
	47, female	L5	1	Hypoplasia	LBP and radiculopathy	Left pedicle defect and right spondylolysis	TLIF with bilateral pedicle screw fixation	1 year

Patients who develop lumbar spondylolisthesis with intractable low back pain and radiating pain should be considered for surgery if conservative treatment is failed (2, 4, 5). Unlike the non-fusion procedures such as isthmus and pedicle repair techniques, which are indeed the choices in the absence of spondylolisthesis, patients with spondylolisthesis should be considered for the solid fusion and fixation of the slipped vertebra with the caudal vertebra due to intersegmental instability. The surgical procedures, including decompression or not, the fusion methods, and the fixation segments, need to be individualized according to the different situations. Accurate screw placement is a major surgical consideration. In patients with pedicle cleft, the posterior part of the pedicle, together with the isthmus and the lamina, is free-floating. The patients with pediculolysis usually manifest hyperplasia and sclerosis, while in patients with hypoplasia, the pedicle is too slender. All the above disadvantages make it hard to achieve precise screw placement into the defective pedicle. Hsieh et al. (3) reported a dysplastic patient treated with anterior lumbar interbody fusion (ALIF) combined with percutaneous unilateral screw placement (i.e., contralateral pedicle to the cleft). However, the fixation strength is weaker than bilateral fixation, and direct nerve root decompression and instrumented reduction of the shifted vertebra cannot be fully achieved. Our experience is that skillful surgeons can still endeavor to the bilateral screw placement of the single segment in these types of patients to improve the effect of fixation and reduction. Meanwhile, the advantages of robotic-assisted trajectory design and execution can be utilized when available (21). Similar to bilateral isthmic spondylolisthesis, radiating pain in the lower limbs in these patients is usually in accordance with the exiting nerve compression. In addition, the symptom may be further exacerbated by hyperostosis and scar formation around the pedicle cleft which forms the superior wall of the foramen. Viswanathan et al. (2) reported a similar case of L5 spondylolisthesis for whom the initial surgery was performed with bilateral single-segmental pedicle fixation. Nevertheless, 1 month after surgery, the patient developed exiting nerve compression symptoms on the side of the pediculolysis. The patient received revision surgery to remove the screw across the pediculolysis, perform medial pediculectomy and L5 root release, and lengthen fixation and fusion to L4 to fulfill solid fixation. From this perspective, a complete decompression of the dorsal aspect of the exiting nerve root and interbody fusion is feasible by a transforaminal approach, which seems to be a more reasonable option. Finally, it is interesting to find that regardless of the causes of the pedicle cleft, bony fusion occurred at both ends of the defect after long-term fixation, which was not mentioned in the previous literature.

In conclusion, lumbar spondylolisthesis with unilateral pedicle cleft and contralateral spondylolysis is rarely reported and can be easily misdiagnosed. There is no widely accepted surgical option for the patients after unsatisfactory conservative treatments. Our experience suggests that good clinical results may be achieved by single-segmental posterior interbody fusion and bilateral pedicle screw fixation. Precise screw placement into the deficient pedicle and sufficient exiting nerve decompression are prerequisites for the success of this surgical option.

## Data Availability

The original contributions presented in the study are included in the article/Supplementary Material; further inquiries can be directed to the corresponding author.
